# Functional Traits Drive Dispersal Interactions Between European Waterfowl and Seeds

**DOI:** 10.3389/fpls.2021.795288

**Published:** 2022-01-31

**Authors:** Bia A. Almeida, Balázs A. Lukács, Ádám Lovas-Kiss, Chevonne Reynolds, Andy J. Green

**Affiliations:** ^1^Department of Wetland Ecology, Doñana Biological Station EBD-CSIC, Seville, Spain; ^2^Wetland Ecology Research Group, Centre for Ecological Research, Institute of Aquatic Ecology, Debrecen, Hungary; ^3^School of Animal, Plant and Environmental Sciences, University of the Witwatersrand, Johannesburg, South Africa; ^4^FitzPatrick Institute of African Ornithology, DST/NRF Centre of Excellence, University of Cape Town, Cape Town, South Africa

**Keywords:** seed dispersal, functional trait, endozoochory, long distance dispersal, waterbirds, Anatidae, migratory birds, feeding behavior

## Abstract

Endozoochory by waterfowl is important for a broad range of angiosperms, most of which lack a fleshy fruit. This dispersal function contributes to the formation and maintenance of plant communities and may allow range shifts for plant species under global change. However, our current understanding of what seed or plant traits are important for this dispersal mechanism, and how they relate to variation in waterbird traits, is extremely limited. We addressed this question using a unique dataset identifying the plant species whose seeds are ingested by 31 different waterfowl species in Europe. We used RLQ and fourth-corner analyses to explore relationships between (1) bird morphological and foraging strategy traits, and (2) plant traits related to seed morphology, environmental preferences, and growth form. We then used Generalized Additive Models to identify relationships between plant/seed traits and the number of waterfowl species that disperse them. Although many waterfowl feed intentionally on seeds, available seed trait data provided little explanation for patterns compared to plant traits such as Ellenberg indicators of habitat preference and life form. Geese were associated with terrestrial plants, ingesting seeds as they graze on land. Diving ducks were associated with strictly aquatic plants, ingesting seeds as they feed at greater depths. Dabbling ducks ingest seeds from plants with high light and temperature requirements, especially shoreline and ruderal species growing in or around the dynamic and shallow microhabitats favored by these birds. Overall, the number of waterfowl vector species (up to 13 per plant species) increases for plants with greater soil moisture requirements and salinity tolerance, reflecting the inclination of most waterfowl species to feed in coastal wetlands. Our findings underline the importance of waterfowl dispersal for plants that are not strictly aquatic, as well as for plants associated with high salinities. Furthermore, our results reveal a soil moisture gradient that drives seed-bird interactions, in line with differences between waterfowl groups in their microhabitat preferences along the land-water continuum. This study provides an important advance in our understanding of the interactions that define plant dispersal in wetlands and their surroundings, and of what plants might be affected by ongoing changes in the distributions of waterfowl species.

## Introduction

Long distance seed dispersal (LDD) events have a vital role in determining plant species distribution, genetic flow among populations and the colonization of unoccupied habitats (Levine and Murrell, 2003; De Queiroz, 2014). Dispersal by animal vectors (zoochory) provides longer distance dispersal in comparison to abiotic vectors (Bullock et al., 2017), with migratory birds able to provide extreme dispersal distances (Popp et al., 2011; Viana et al., 2016; Viana, 2017). These birds mainly disperse seeds *via* gut passage (endozoochory), yet their importance for plants lacking a fleshy-fruit has been underestimated because it is ignored by widely used “dispersal syndromes” (Green et al., 2021). Many of the migratory bird species are waterbirds, which are important for the dispersal of a broad range of both aquatic and terrestrial plants between habitat patches, often facilitating dispersal at extreme distances of >100 km (Green et al., 2016, 2021; Viana et al., 2016; Martín-Vélez et al., 2021). The waterbird group best known for seed dispersal is that of the waterfowl (Anatidae: ducks, swans and geese) (Green et al., 2016, 2021; Soons et al., 2016). These birds are well-studied partly due to their exploitation for hunting (Green and Elmberg, 2014), and many species are abundant long-distance migrants (Wetlands International, 2021). Waterfowl are vital vectors for maintaining connectivity between plant populations in wetlands lacking hydrological connections (Amezaga et al., 2002).

Food selection and ingestion are the first stages of seed dispersal and ingestion is the main component of dispersal “quantity” (Schupp et al., 2017). Plant and seed traits, as well as vector traits, may influence seed uptake, so identifying these traits can provide insights as to the mechanisms behind plant community establishment (Schleuning et al., 2020). Interactions between plants and frugivorous birds (i.e., those feeding on fleshy-fruits) in terrestrial ecosystems are known to depend on many traits of both groups of organisms, such as fruit size and palatability and bill morphology (Wenny et al., 2016; Donoso et al., 2017). On the other hand, despite recent advances in identifying the interactions between different waterfowl and plant species in a particular community (Reynolds and Cumming, 2016; Sebastián-González et al., 2020; Silva G. G. et al., 2021), the trait associations underlying the uptake of seeds by different waterfowl species remain unknown. In general, seed ingestion by non-frugivorous vertebrates is not size limited in a manner comparable to frugivores (Chen and Moles, 2015; but see Gurd, 2008).

Finding relationships among traits of seed and waterfowl species may be complicated by intraspecific plasticity and variation. Waterfowl sometimes select seeds or fruits individually, but usually ingest seeds collectively while filtering food items out of water or sediments, while ingesting other plant parts, or even ingest them secondarily when eating animal prey (Goodman and Fisher, 1962; Li and Clarke, 2016; Pecsics et al., 2017). A given waterfowl species may use more than one foraging substrate and different foraging strategies (Li and Clarke, 2016), and encounter a diversity of habitats and flora along their migration routes. On the other hand, aquatic plants have different growth forms and are often amphibious, while terrestrial plants can occur in moist soils where seeds can be ingested by grazing waterfowl, or their seeds may be washed or blown into waterbodies (Wells and Pigliucci, 2000; Lovas-Kiss et al., 2019; Li et al., 2020). Seeds of terrestrial plants also become available to waterfowl during seasonal floods. Nevertheless, waterfowl are traditionally placed into different foraging guilds, and their habitat use (e.g., in relation to depth and shoreline vegetation) is considered to be related to body size and bill morphology (Nudds et al., 2000; Guillemain et al., 2002; Kear, 2005; Gurd, 2008). Therefore, we expect differences in foraging niche between waterfowl species to be reflected in differences in the traits of the plants they disperse.

For the present study, we have established a comprehensive dataset on the plant seeds ingested by European waterfowl. Although there is more information about waterfowl diet in North America, we focus on Europe because of the quality of information on plant traits. Our dataset includes a mixture of data demonstrating seed ingestion from the upper and lower digestive tracts, as well as from the feces of individual birds. The presence of seeds in the upper digestive tract of waterfowl has been shown to be a good proxy for seed dispersal by endozoochory (Kleyheeg et al., 2015; Soons et al., 2016; Costea et al., 2019). Previous studies have used part of this same dataset regarding seven dabbling duck species, including a comparison of traits between European plants whose seeds were ingested, and plants whose seeds were not (Dessborn et al., 2011; Soons et al., 2016).

Here, we investigate if different waterfowl species (including ducks, geese, and swans) tend to disperse plants with different traits. We evaluate how seed and other plant traits determine their chances of dispersal in two different ways. Firstly, to assess whether some plant species have a higher chance of being transported by a particular waterfowl species, we assess whether duck, geese and swan traits act as filters to determine the traits of the seeds they ingest. Then, to evaluate if there are plant characteristics that give them higher chances of being transported, we analyze how seed and plant traits influence the number of different waterfowl species that disperse each plant species.

## Materials and Methods

### Diet Data

We conducted a systematic search on all information on the presence of angiosperm diaspores (hereafter called seeds) in the alimentary canal or feces of European waterfowl and used this as a proxy for dispersal interactions. As a starting point, we took the studies for dabbling ducks compiled by Dessborn et al. (2011) and Soons et al. (2016), together with the accounts for all waterfowl species in Snow et al. (1998). We then added further studies for all waterfowl, by searching in Google Scholar and in the Web of Science up to March 20, 2021. We used as keywords “gut content,” “dietary data or diet,” and “fecal data or fecal samples” together with the scientific name of each species. We included in our search all 39 waterfowl species that occur in Europe (as both wintering or breeding species) according to the IOC World Bird List (Gill et al., 2021). For species where scientific names have changed (e.g., changes in genera from *Anas* to *Mareca* or *Spatula*) we included all known versions of the name. A list of the literature we used in our study is found in Supplementary Material 1. Furthermore, we also used unpublished data from the authors’ recent studies of seed dispersal *via* fecal analysis.

We considered only diet data from adult waterfowl and only the presence of seeds from a given plant species in the diet of the waterfowl species, as abundance or frequency data are often unreported, or are not quantified in a comparable manner. As we were looking for trait-dependent relationships, seeds from 85 plant taxa that were not identified to the species level were excluded from further analyses. Domestic plant species (e.g., rice or barley) were also removed from further analyses since they are often used to bait waterfowl, and are less likely to be dispersed (Lovas-Kiss et al., 2018). We did not exclude non-native plants or bird species. From this database, we produced a waterfowl by plant species matrix indicating the occurrence of each seed species in the diet of each waterfowl species (Supplementary Material 2). We were unable to account for spatial or temporal factors due to limitations of our technique of analysis and to the low number of studies found for many of the bird species (Table 1).

**TABLE 1:** Anatidae species for which seed ingestion data was found, their feeding groups (i.e., guilds), and information on the numbers of published studies found in our literature search (see Supplementary Material 1 for details), unpublished datasets used, locations for which data were found, sampled individuals, and plant species whose seeds were ingested by each bird species.

Species	Feeding group	Number of published studies	Number of unpublished datasets	Number of sampled locations	Number of sampled individuals	Number of sampled droppings	Number of plant species found in diet
*Anas acuta*	OmDab	13	4	13	438	95	76
*Anas crecca*	OmDab	24	6	21	2,997	57	213
*Anas platyrhynchos*	OmDab	32	7	31	2,999	431	240
*Anser albifrons*	TerPl	1	0	1	260	0	3
*Anser anser*	TerPl	3	2	5	0	74 + 5 kg	132
*Anserbrachyrhynchus*	TerPl	0	0	0	0	0	2
*Anser erythropus*	TerPl	3	0	3	203	720	9
*Anser fabalis*	TerPl	1	0	1	175	0	5
*Aythya ferina*	OmDiv	7	1	7	141	61	38
*Aythya fuligula*	OmDiv	6	0	7	431	0	40
*Aythya marila*	OmDiv	2	0	2	463	0	6
*Aythya nyroca*	OmDiv	2	0	2	2	0	3
*Branta canadensis*	TerPl	1	0	1	0	50	6
*Branta leucopsis*	TerPl	0	1	2	0	45	10
*Bucephala clangula*	OmDiv	5	0	6	201	0	24
*Bucephala islandica*	OmDiv	1	0	1	48	0	1
*Clangula hyemalis*	MarInv	2	0	2	528	0	2
*Cygnus columbianus*	AqPl	2	0	2	60	0	8
*Cygnus cygnus*	AqPl	0	1	1	0	77	1
*Cygnus olor*	AqPl	1	0	1	8	0	7
*Mareca penelope*	OmDab	12	3	13	543	140	75
*Mareca strepera*	OmDab	9	2	8	475	38	42
*Marmaronetta angustirostris*	OmDab	5	0	3	105	52	5
*Melanitta nigra*	MarInv	1	0	1	250	0	3
*Mergus serrator*	FiEat	1	0	1	143	0	4
*Netta rufina*	OmDiv	3	0	2	1	8	1
*Oxyura leucocephala*	OmDiv	0	1	1	17	0	5
*Spatula clypeata*	OmDab	11	2	10	365	109	46
*Spatula querquedula*	OmDab	4	0	3	213	0	31
*Somateria mollissima*	MarInv	1	0	1	50	0	1
*Tadorna tadorna*	OmDab	6	1	6	663	147	10
Total		63	30	68	11,820	2,104	407

*OmDab, Omnivorous dabbling ducks; OmDiv, Omnivorous diving ducks; TerPl, Terrestrial plant-eaters; AqPl, Aquatic plant-eaters; MarInv, Marine invertebrate-eating diving ducks; FiEat, Fish-eating diving ducks.*

### Waterfowl Traits

For both plant species and waterfowl species, we compiled available information on traits that we considered to be possible drivers of the interaction between waterfowl and seeds (Supplementary Material 3). Available trait information for waterfowl included morphological measurements and feeding guilds. Waterfowl morphological measurements of bill length (from tip to skull along the culmen, and from tip to nares), beak width and depth at the nares and tarsus length were provided by Pigot et al. (2020). Estimates of mean body mass (g) were sourced from Wilman et al. (2014). Body mass can be a proxy for species’ size and, together with tarsus length, may indicate the depth or height reached by a particular species. On the other hand, beak morphology may be associated with differences in seed uptake at a given feeding depth/height (Poysa, 1983a; Kear, 2005). Based on previous studies on waterfowl diet and foraging behaviors (Kear, 2005), we assigned ducks, geese and swans into six feeding guilds: Omnivorous dabbling ducks (OmDab from hereon), Omnivorous diving ducks (OmDiv), Terrestrial plant-eaters (TerPl), Aquatic plant-eaters (AqPl), Marine invertebrate-eating diving ducks (MarInv), and Fish-eating diving ducks (FiEat). In most studies where seeds were recorded in their diet, MarInv were feeding in low salinity habitats such as Lake Myvatyn in Iceland, or the Baltic Sea, where they co-occurred with OmDab and OmDiv.

### Plant/Seed Traits

For plants, we considered traits describing environmental preferences, growth form and seed morphology. We sourced Ellenberg Indicator Values (EIVs, Ellenberg and Leuschner, 2010) for soil moisture (F), nutrients (N), salinity (S), temperature (T), and light exposure (L). EIVs indicate the general preference of a plant species for specific environmental conditions, and thus may vary geographically for each plant species with extensive ranges, according to climatic variation. Hence to avoid bias, where possible we used EIV values from the LEDA database (Kleyer et al., 2008). When values for a given species were not available in LEDA, we retrieved them from one of the following sources: PLANTATT (Hill et al., 2004), Pladias database (Chytrý et al., 2021), Baseflor (Julve, 1998), and TRY database (Kattge et al., 2020). Seed morphology is likely to influence the rates at which waterfowl ingest seeds (Guillemain et al., 2002; Green et al., 2016; Lovas-Kiss et al., 2020a). Seed morphological traits used were seed mass (g), seed size (mm^3^), seed density (g/mm^3^), and seed roundness. We did not use morphological dispersal syndromes because they are not relevant to endozoochory by waterfowl (i.e., the great majority of plants dispersed by endozoochory do not have an “endozoochory syndrome,” Green et al., 2021). Seed size was estimated as a multiplication of seed length (Le), width (Wi), and thickness (Th). Seed density was estimated as seed mass divided by seed size, and seed roundness as (Wi/Le + Th/Le + Th/Wi)/3. Morphological values were sourced from the same databases used for EIVs. Seed mass was retrieved from the Seed Information Database of Kew Gardens (Royal Botanic Gardens Kew, 2021). We categorized plant species according to the most basic life form as: terrestrial, helophyte, hydato-helophyte (i.e., helophyte species that can have a submerged form), hygrophyte (i.e., amphibious, hydrophyte species that have terrestrial form), and hydrophyte. Finally, we classified hydrophytes, hygrophytes and helophytes into the main growth forms, according to the most typical zone occupied by each species in the aquatic environment. For this, we created four non-exclusive binary variables: submerged plant, floating leaved plant, emergent plant and wet soil plant (Supplementary Material 4). Since our analytical methods do not permit missing values, missing plant trait values (2.1%) were substituted for arithmetic means of all values observed for the corresponding trait.

### Data Analysis

To assess whether some plant species have a higher chance of being transported by particular waterfowl species, we used RLQ (Dolédec et al., 1996) and fourth-corner analyses (Dray et al., 2014). RLQ has traditionally been recommended to evaluate univariate relationships between environmental variables and species functional traits (Almeida et al., 2018; Schack et al., 2020; Silva J. L. A. et al., 2021). We have utilized the method to look instead at the relationships between waterfowl species traits (substituting environmental variables) and plant species functional traits. In this way, we can ask how waterfowl functional traits might act as filters on plant traits, thus influencing seed dispersal processes and community sorting.

We followed the RLQ protocol using the package *ade4* (Dray and Dufour, 2007), in the R environment, version 4.0.4 (R Core Team, 2021). Correlations between both trait tables and the axes of the RLQ ordination were calculated to evaluate which traits were best represented by the RLQ axes. For the first two axes of the RLQ, we considered those trait-axis correlations exceeding 0.5 to be important. We used three approaches to test waterfowl trait x plant trait associations. First, we tested the overall significance of the RLQ analysis. Next, we tested the significance of direct trait-trait relationships directly on the raw data tables. Finally, we tested for the significance of the trait-axis relationships, in which those traits correlated with the same axis can be interpreted as associated traits. We used 49,999 permutations and permuted both waterfowl and plant species as a procedure to test for significance (α = 0.05) in all three tests, and adjusted *P*-values for multiple testing using the false discovery rate method (FDR, Benjamini and Hochberg, 1995). See Supplementary Material 5 for further details on the RLQ and fourth-corner protocol.

To determine if particular seed/plant traits are preferred by a greater number of waterfowl species, we used zero-truncated Generalized Additive Modeling (GAM). GAMs use smoothing curves to model relationships between variables. As GAM allows both linear and non-linear fits, it is very useful for modeling data with non-linear distributions (Zuur et al., 2007). We used zero-truncated models because our data represented counts (number of bird species associated with a given plant species), and only plant species whose seeds were ingested by at least one waterfowl species were included (Zuur et al., 2009). We fitted models of the number of waterfowl species that consumed each seed species (hereafter number of vector species) against the predictor variables of soil moisture, nutrient, salinity, temperature, and light exposure EIVs, and seed roundness, mass and density. We did not include seed volume because this was highly correlated with seed mass (Pearson’s *r* = 0.78). We also excluded categorical and binomial variables describing plant life forms, because these were related to soil moisture (EIV F). Seed roundness, mass and density were natural log transformed prior to model construction to reduce the effect of extreme values. The relationships of temperature, light exposure and moisture EIVs with the response variable were assumed to be linear and modeled as such (Supplementary Table 8). We modeled the number of vector species against the predictor variables with the negative binomial error distribution to account for overdispersion. We fitted models with all possible combination of factors, and used the Akaike Information Criteria (AIC) for model selection. As we had many models with similar ΔAIC values, we considered as the best model to be the one with the lowest number of terms and ΔAIC <6 (Zuur et al., 2007). We plotted the deviance residuals against the explanatory variables, and compared the observed and fitted counts in a rootogram (a modified histogram plotting the square roots of frequencies) as part of model validation. We fitted our models using the function *vgam* from the package *VGAM* (Yee, 2020), in the R environment, version 4.0.4 (R Core Team, 2021).

## Results

Studies containing seed ingestion information were sourced for 31 waterfowl species across Europe. The number of individuals and sites sampled varied widely among bird species and, in general, dabbling ducks (OmDab) were better sampled than other waterfowl species (Table 1). After removing the plants whose seeds were identified only to family or genus level (*n* = 85) and domesticated species (*n* = 16), 407 plant species were recorded as being ingested by at least one bird species. The mean number of vectors ingesting each plant species was 2.58 (s.e. = 0.12), with 49.4% of the plant species being ingested by only one vector, and a maximum of 13 vectors ingesting one plant species (for the sea club-rush *Bolboschoenus maritimus* and the mare’s tail *Hippuris vulgaris*).

### RLQ Analysis

**FIGURE 1:**
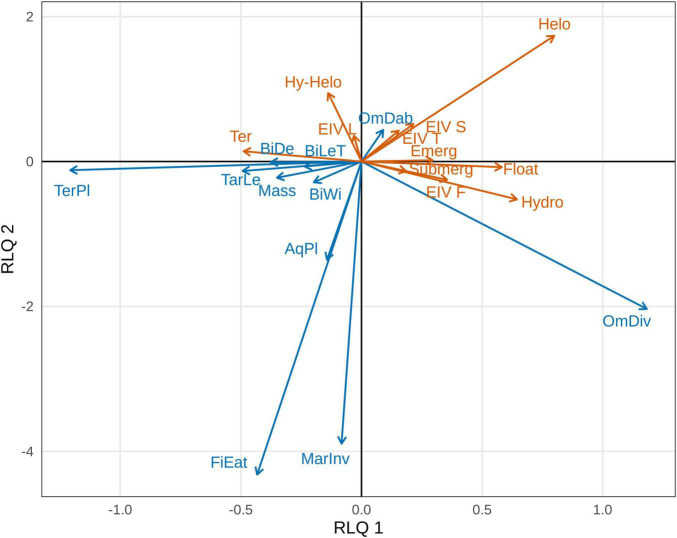
Waterfowl (in blue) and plant traits (in orange) projected onto the first two axes of the RLQ analysis. We only present traits with correlations >0.5 with at least one axis. Traits showing positive (or negative) associations to the same axis are interpreted as being associated. Values of the correlations of each trait to each axis can be found in Supplementary Material 6, and their significance according to the fourth-corner test is given in Figure 3. BiLeT, Bill length; BiDe, Bill depth; BiWi, Bill width; TarLe, Tarsus length; OmDab, Omnivorous dabbling ducks; OmDiv, Omnivorous diving ducks; TerPl, Terrestrial plant-eaters; AqPl, Aquatic plant-eaters; MarInv, Marine invertebrate-eating diving ducks; FiEat, Fish-eating diving ducks; EIV, Ellenberg Indicator Value for soil moisture (F), salinity (S), temperature (T), and light exposure (L); Helo, Helophyte; Hy-helo, Hydato-helophyte; Hydro, Hydrophyte; Ter, Terrestrial; Submerg, Submerged; Float, Floating leaved; Emerg, Emergent.

The first two axes of the RLQ analysis explained 80.76 and 10.93% of the total co-inertia, respectively, and were retained for further analysis (Supplementary Material 5). Considering only correlation values above 0.5, the first RLQ axis was positively correlated with the foraging guilds marine invertebrate-eating diving ducks (MarInv) and omnivorous diving ducks (OmDiv), and with the Ellenberg indicator value for soil moisture (EIV F), helophytes, hydato-helophytes and hydrophytes and with the submerged, floating leaved and emergent growth forms. This same axis showed negative correlations with the following bird traits: bill length, width and depth, tarsus length, body mass, aquatic plant-eaters (AqPl) and terrestrial plant-eaters (TerPl), and with the terrestrial life form of plants. The second axis presented positive correlations with the foraging guild omnivorous dabbling ducks (OmDab), with EIVs for salinity (S), temperature (T), and light (L), and with helophytes. This axis also showed negative correlations with bill width, tarsus length, body mass, the foraging guilds fish-eating diving ducks (FiEat), AqPl, MarInv, OmDiv, and TerPl and with the hydrophyte life form (Figure 1 and Supplementary Material 6).

### Fourth-Corner Tests

**FIGURE 2:**
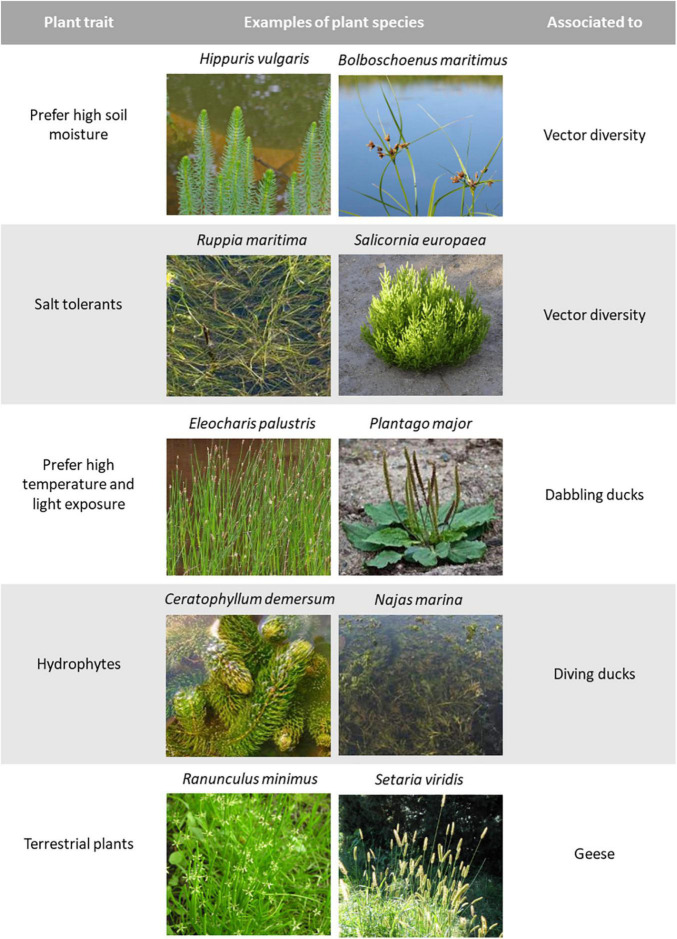
Plant functional traits involved in the main associations found in our RLQ analysis and GAM. We show examples of plant species that present each trait and with what aspect of waterfowl vectors each trait was associated with. Trait values, waterfowl species that ingest seeds of each plant species and credits for the photographs can be found in Supplementary Materials 2, 3, 4, 8.

**FIGURE 3:**
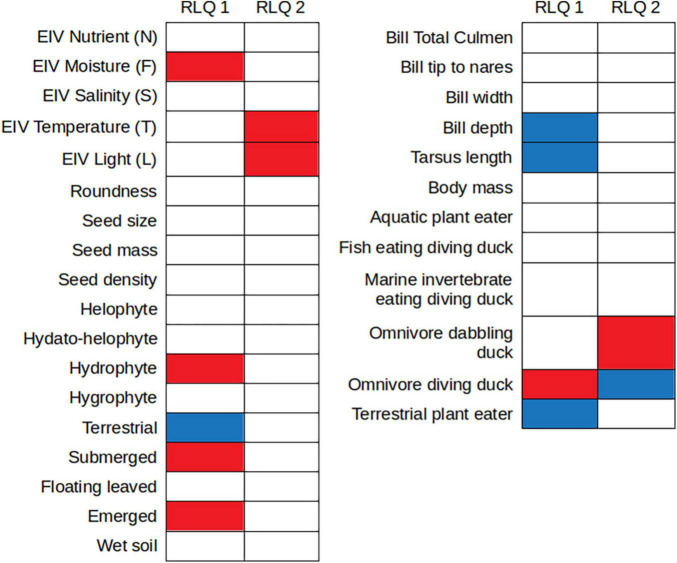
Results of the fourth-corner tests on the associations of each plant and waterfowl trait with the first two axes of the RLQ. Blue and red cells represent, respectively, positive and negative significant associations. White cells represent associations not considered as significant by the fourth-corner tests. These results indicate associations between geese species and terrestrial plants, between omnivorous diving ducks and strictly aquatic plants, and between dabbling ducks and plants that prefer higher temperature and light exposure.

The general permutation test showed that there was a statistically significant global link between plant and waterfowl traits, according to the permutations of plant species (*p* < 0.001) and waterfowl species (*p* = 0.046). Furthermore, the fourth-corner tests found significant correlations for both trait-trait and trait-axis relationships. According to the trait-trait test, bird species belonging to the TerPl foraging guild were negatively correlated with soil moisture EIV (i.e., these waterfowl species interacted more with species requiring low soil moisture, see Figure 2), while those belonging to the OmDiv guild were positively correlated with submerged plants (i.e., omnivorous diving ducks interact more with seeds of submerged macrophytes, see Figure 2). Furthermore, the trait-axis test found that the first RLQ axis showed significant negative correlations with bird tarsus length, bill depth, body mass, with the foraging guild TerPl, and with plants with the terrestrial growth form. This depicts a relationship between geese species and terrestrial plants (Figures 1–3). This same axis showed significant positive correlations with the foraging guild OmDiv, with soil moisture EIV, with plants with the hydrophyte life form, and with submerged, floating leaved and emergent plants. This reflects a relationship between omnivorous diving ducks and strictly aquatic plants in all their growth forms (Figures 1–3). The second RLQ axis was significantly negatively correlated with OmDiv, but not with plant traits, and significantly positively correlated to OmDab and with the light and temperature EIVs, representing a relationship between dabbling ducks and plants that prefer higher temperature and light exposure (Figures 1–3).

### Fitted Generalized Additive Modeling

**FIGURE 4:**
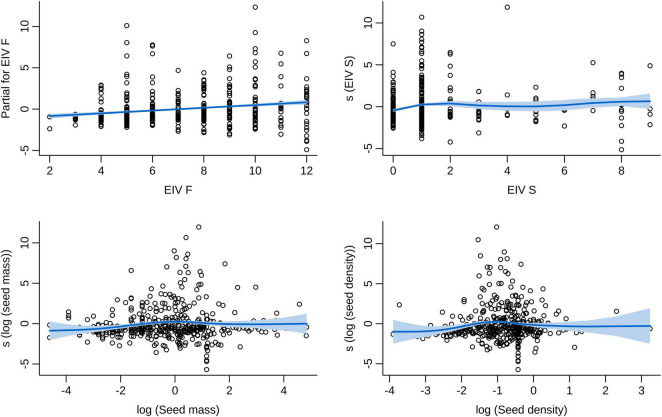
Relationship between plant/seed traits and the number of avian vector species. Log seed mass and log seed density presented unimodal relationships with the number of vector species. The soil moisture EIV was positively and linearly related with the number of vector species, and the salinity EIV had a non-linear relationship with the number of vector species. Fitted lines and their standardized error bands represent the final zero-truncated GAM models. The *Y* Axis represents centered partial residuals from the model fit (the “s” term represents smoothing terms of the variables modeled as non-linear). EIV, Ellenberg Indicator Value for soil moisture (F) and salinity (S). Seed mass and seed density are weakly correlated (Spearman’s ρ = 0.18, *p* < 0.001).

In our best model (the one with the lowest number of terms and ΔAIC < 6), the seed traits of mass and density, and the EIVs for soil moisture and salinity (F and S) were significant predictors of the number of vector species dispersing each plant species (Supplementary Material 7). Smoothing terms indicated that log seed mass and log seed density presented unimodal relationships with the number of vector species (Figure 4). However, both ends of the smooth line presented very large standardized errors (Figure 4), due to the low number of plant species with extremely high or low mass and density values (even after data transformation). The soil moisture EIV smoother revealed a positive linear relationship between moisture and the number of vector species (Figures 2, 4). The salinity EIV smoother revealed an increase in the number of vector species from values 0 to 2, a slight decrease from there to value 5, followed by a slight increase from then on (Figures 2, 4).

## Discussion

We found evidence that waterfowl traits and seed and other plant traits interact to act as filters for plant dispersal *via* endozoochory. A unique dataset on seed ingestion by European Anatidae species allowed us to elucidate patterns in trait-trait relationships between the two groups, in the first study of its kind. We found that seed ingestion differs between waterfowl groups in a manner closely associated with differences in their selection of foraging habitat (Kear, 2005). This is particularly clear for herbivorous geese that feed on terrestrial plants and ingest seeds of more terrestrial species, most likely together with green plant material in a manner consistent with the “foliage is the fruit hypothesis” (Janzen, 1984; Green et al., 2016, 2021). At the other extreme, omnivorous diving ducks are typically bottom feeders in deep water, and tend to ingest the seeds of hydrophytes, whatever their growth form. In between are the dabbling ducks associated with shoreline habitats that are typically dynamic with high rates of disturbance, where they consume seeds of plants that prefer higher light exposure and temperature. We also investigated the plant traits that favor seed consumption by a greater number of waterfowl species and found that both higher moisture requirements and greater salinity tolerance favor seed ingestion by more waterfowl species. Seeds of intermediate mass and density (on a log scale) were also ingested by more waterfowl species.

### Importance of the Moisture Gradient

**FIGURE 5:**
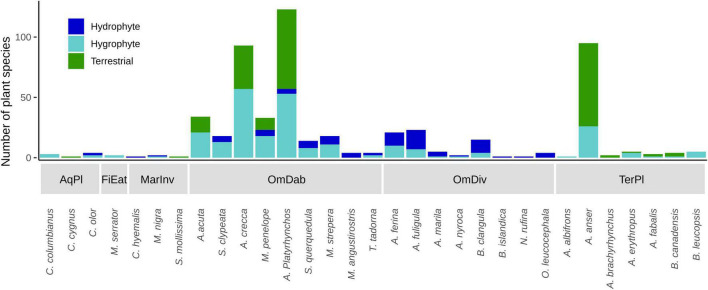
Number of hydrophyte, hygrophyte and terrestrial plant species whose seeds are known to be ingested by each bird species in each feeding group. AqPl, Aquatic plant-eaters; FiEat, Fish-eating diving ducks; MarInv, Marine invertebrate-eating diving ducks; OmDab, Omnivorous dabbling ducks; OmDiv, Omnivorous diving ducks; TerPl, Terrestrial plant-eaters. See Table 1 for full names of bird species.

Waterfowl feeding groups ingest seeds of plants with different life forms along the soil moisture gradient, with geese and omnivorous diving ducks at the terrestrial and strictly aquatic extremes, respectively (Figure 2). Other feeding groups were not associated with this gradient, although this may be due to limited data and diet literature in the case of fish-eating ducks FiEat, marine ducks MarInv and swans AqPl, which presented a very low number of interactions (see Table 1 and Figure 5). Dabbling ducks were the best sampled group in our dataset, and they ingested seeds from plants found along the whole moisture gradient (see Figure 5). This suggests that dabbling ducks (exemplified by the ubiquitous mallard *Anas platyrhynchos*) play a more generalist role in the dispersal of different plant life forms, feeding in different habitats with a range of foraging strategies (see also Soons et al., 2016).

### Light and Temperature Requirements of Plants Dispersed

Our RLQ analysis indicated that dabbling ducks are more likely to ingest seeds of plants that prefer higher temperature and light exposure. This result is consistent with Soons et al. (2016), who found that dabbling ducks are more likely to disperse plants that prefer higher light exposure than those which don’t (a different analysis using part of this same dataset, but comparing it with European angiosperms for which dispersal by ducks has not been recorded). This suggests our result is unlikely to be a consequence of sampling bias, even though waterfowl species were unevenly sampled between latitudes and seasons. A preference of dabbling ducks for ingesting seeds from warmer, more open microhabitats likely reflects their affinity for exposed shoreline habitats and shallow, temporary wetlands such as seasonally flooded grasslands with short hydroperiods (Kear, 2005). Such habitats are created or maintained in response to some extent of stress and disturbance (e.g., hydrological variation, livestock, or urbanization) that do not allow aquatic or terrestrial vegetation to reach a mature late successional stage (Grime, 1977). For this reason, they are typically occupied by annual shoreline or ruderal plant species with high light and temperature preferences.

### Different Waterfowl Have Different Roles as Seed Vectors

Our results for ingestion of seeds from plants with different traits confirm that different waterfowl species vary in their roles as seed dispersers. This is consistent with previous studies in South Africa and Brazil (Reynolds and Cumming, 2016; Silva G. G. et al., 2021) showing that differences in the species composition of seeds dispersed by waterfowl species are related to their foraging behavior (note, these studies did not consider plant traits). In contrast, a study of other waterbirds in rice fields found no difference between a gull and stork species in the plants they dispersed (Martín-Vélez et al., 2021). Ours is the first study showing that both bird and plant traits drive waterfowl-seed interactions. Our trait-trait analyses showed that amongst waterfowl, feeding group was the trait with the strongest association with seed and other plant traits. Different waterfowl species also vary greatly in their migration and other movement patterns (Scott and Rose, 1996; Wetlands International, 2021), a major driver for long-distance seed dispersal (LDD) patterns. Hence, ongoing changes in abundance and migration patterns of many waterfowl species (Ramo et al., 2015; Pavon-Jordan et al., 2019) will have direct implications for plant dispersal and distributions, although this is outside the scope of the present study.

### Plant and Seed Traits That Favor a Diversity of Vectors

Seeds of plants with a preference for higher soil moisture and salinity tend to be ingested and dispersed by more waterfowl species. Even waterfowl that can feed on land will also feed in aquatic habitats (e.g., geese feed in shallow marshes or ricefields, Snow et al., 1998), and so aquatic seeds are those most likely to be encountered by a wider range of vectors. The salinity effects are consistent with general patterns of biogeography and habitat use by migratory waterfowl, as these birds concentrate particularly in larger, low altitude wetlands in coastal plains and deltas that tend to have higher salinity than the smaller, higher altitude habitats holding the highest diversity of aquatic plants and invertebrates (Guareschi et al., 2015; MWO, 2018). Among inland wetlands, salinity is naturally high in closed-basin lakes that are widespread waterfowl habitat in southern Europe, and has increased through water extraction for agriculture and urban use in many areas (Jeppesen et al., 2015; MWO, 2018). Freshwater habitats of lower salinity support a greater diversity of aquatic plant species, but have been destroyed by human activities faster than wetlands of higher salinities (Green et al., 2002).

We also found that more bird species dispersed plants with intermediate values of seed mass and density. As seed mass and density were logged before model construction, these intermediate values were actually closer to the lightest and lowest density seeds than to the heaviest and densest. These results are consistent with differences between the seed size distributions of European angiosperms dispersed by dabbling ducks and those which are not (Soons et al., 2016). Owing to volumetric constraints, more seeds are ingested in a single waterfowl meal as seed size decreases (Mueller and van der Valk, 2002). On the other hand, waterfowl themselves are expected to ignore tiny seeds because of mechanical constraints in their filtering apparatus, and the difficulty of separating food items from unwanted sediments and detritus (Gurd, 2008). Hence, it is not surprising to find that birds are more likely to ingest intermediate-sized seeds, but this does not itself demonstrate selection based on size. Furthermore, we cannot rule out the possibility that the smallest seeds have been underestimated in waterfowl guts because diet studies in the literature may have sometimes ignored them. Since our models of the number of waterfowl species that consumed each plant species are based not on the total diversity of plants that exist in the sampled localities, but instead on the set of plants that have been found in waterfowl diet, it is also possible that the birds tend to ingest intermediate-sized seeds because these plant species might be relatively more abundant than others (an explanation that might also apply for seed density). In principle, waterfowl might be more likely to ingest seeds of intermediate density because those of higher density may be less likely to float or even reach the surface of water where they are most readily ingested, whereas those of even lower density may be of little energetic value as avian food.

### Potential Importance of Differences Between Bird Species Within Feeding Groups

Our analysis depended on the placement of waterfowl species into feeding groups. However, we recognize that within feeding groups, different waterfowl species may also have nuanced seed dispersal roles. The variation of particular traits within each feeding group is likely to differentiate species, and may drive species-specific foraging preferences or capacities. The best evidence for this comes from dabbling ducks, where there are interspecific differences within a given locality in the identity of seeds consumed (Brochet et al., 2012) and in how they use their habitat (Green, 1998; Nudds et al., 2000; Arzel and Elmberg, 2004). For example, smaller Eurasian teal *Anas crecca* are more limited in the depth range at which they can extract seeds from sediments than the larger mallard, and also select more small-seeded species (Guillemain et al., 2002). Different traits should be important within each feeding group, as foraging techniques differ between groups. An example of a trait that can be important within a particular group is the density of bill lamellae, which is related to seed size selection in filter-feeding ducks. A higher lamellar density reduces the costs of filtering of smaller items and explains e.g., why Eurasian teal ingest smaller seeds than mallards (Guillemain et al., 2002; Gurd, 2008; Brochet et al., 2012).

### Implications for Seed Dispersal Interactions at Different Geographical Scales

Foraging niche separation in waterfowl has previously been shown to partly depend on general food availability, with birds adapting their feeding strategies according to ecosystem productivity, the number of species and/or individuals sharing the same wetland, and seasonal changes in resource availability such as when ducks deplete available seeds gradually during the winter (DuBowy, 2000; Pöysä and Sorjonen, 2000; Guillemain and Fritz, 2002; Tinkler et al., 2009). Niche separation can be reduced at a local scale when food is more available (Pöysä, 1983b; DuBowy, 1988; Guillemain and Fritz, 2002). Our dataset has low temporal and spatial resolution, and is based on data collected in different types of habitats sampled in different seasons. The trait-trait associations found here at a continental scale may not necessarily be reproduced at a local scale, such as in a given wetland ecosystem where interaction networks represent communities subjected to specific conditions of food availability, competition and seasonality (see e.g., Sebastián-González et al., 2020).

Unfortunately, we were unable to control for latitude or season in our analyses due to imprecision and low sample sizes of the available data, and to technical limitations of the RLQ and fourth-corner methods, which currently do not allow the use of continuous covariables. Although our dataset includes all the diet studies we could find on Anatidae species in Europe, it is likely that the actual number of plant species dispersed is underrepresented, even for well-studied bird species. This is suggested by the large fraction of seeds not identified to species level, by major differences in plant community composition between different sites where waterfowl were sampled (Green et al., 2016; Sebastián-González et al., 2020), and by the continuous increase in total number of plant species detected as the number of individual birds analyzed at a given site increases (Soons et al., 2016).

We necessarily adopted a general approach considering only the presence of interaction events, without considering the frequency with which these interactions occurred or separating data by habitat type or seasons. Furthermore, there is a large variability in sampling effort between bird species and feeding groups. Nevertheless, our analyses captured important relationships between the traits of plant and waterfowl species present in Europe. If we had been able to incorporate differences in seed abundance in the diet of each bird species, and if all bird groups were better represented in our dataset, we may have found more and stronger relationships between traits of both groups. In order to improve our capacity to perform future analyses, more sampling of seed dispersal is required, especially to cover the additional eight waterfowl species in Europe that are not represented in this study (due to absence of data), and to provide more data from the spring migration and summer periods. Non-destructive fecal sampling is an ideal method for this (Hattermann et al., 2019; Lovas-Kiss et al., 2019).

## Conclusion

Seeds have a great diversity of architecture that allows them to survive gut passage, and optimality models explain why waterfowl generally digest only part of their food before egestion (Van Leeuwen et al., 2012; Costea et al., 2019). Even a fraction of soft fish eggs can survive gut passage (Lovas-Kiss et al., 2020b). Therefore, for any plant species a fraction of seeds ingested by waterfowl survives gut passage, with the possible exception of exceptionally large seeds (Brochet et al., 2009; Soons et al., 2016). Thus, our analysis of how plant and waterfowl traits influence seed ingestion can be understood as a good proxy for which seeds are most probably dispersed by which waterfowl species.

Darwin (1859) famously suggested that “the widespread distribution of fresh-water plants … depends on the wide dispersal of their seeds by fresh-water birds,” although he emphasized epizoochory as the mechanism and overlooked their role in endozoochory (Green et al., 2016). Although modern literature continues to repeat the idea that waterbirds are only important for dispersing aquatic plants (e.g., Viana, 2017; González-Varo et al., 2021), our findings emphasize their importance as vectors for many non-aquatic plants, as well as for plants of saline or brackish waters.

The ingestion of seeds by waterfowl, the first step in the endozoochory process, depends on both plant and bird species traits. Our results suggest that the main mechanism through which duck, geese and swan traits filter the seeds they consume is seed availability in their foraging habitat. Even though we pooled data across seasons, waterfowl feeding groups differed in their function as seed dispersers. Given the exploratory nature of our analysis, our findings need to be tested with other datasets, e.g., from other biogeographical areas. For example, improved plant trait databases are needed for North America (where there is no accessible equivalent to many traits used in our analyses), where waterfowl diet data are particularly extensive (Baldassarre and Bolen, 2006; Callicut et al., 2011). Even for Europe, more extensive data are still required for other traits likely to be important as determinants of ingestion by waterfowl and seed survival during gut passage. Such traits include seed hardness (Lovas-Kiss et al., 2020a) and are also likely to include seed chemistry such as chemical defenses, nutritional content (Petrie, 1996; Dugger et al., 2007), and seed color (Green et al., 2016, 2021).

We hope our study will inspire more research into the importance of seed traits for non-classical endozoochory (i.e., endozoochory by non-frugivorous animals, Green et al., 2021). Waterfowl facilitate LDD events that allow plants to keep pace with climate change (Viana, 2017). Our study provides an important step toward predicting which plants might be affected by changes in the distribution of particular waterfowl species. Further research in seed dispersal by waterbirds should approach how species traits drive seed-bird interactions locally, how these interactions vary through time due to changes in diet and behavior, and how much waterfowl food preferences vs. seed availability influences seed ingestion and dispersal (see also Callicut et al., 2011; Green et al., 2021 for research needs).

## Data Availability Statement

The original contributions presented in the study are included in the article/Supplementary Material, further inquiries can be directed to the corresponding author.

## Author Contributions

BA, AG, and CR conceived the idea. BA, ÁL-K, and BL collected the seed ingestion and trait data. BA analyzed the data and wrote the first draft. All authors participated in discussing the idea, revising and approving the final manuscript.

## Conflict of Interest

The authors declare that the research was conducted in the absence of any commercial or financial relationships that could be construed as a potential conflict of interest.

## Publisher’s Note

All claims expressed in this article are solely those of the authors and do not necessarily represent those of their affiliated organizations, or those of the publisher, the editors and the reviewers. Any product that may be evaluated in this article, or claim that may be made by its manufacturer, is not guaranteed or endorsed by the publisher.
