# Supplementary material for: Functional Traits Drive Dispersal Interactions Between European Waterfowl and Seeds
Source: Front Plant Sci. 2022 Jan 31;12:795288. doi: 10.3389/fpls.2021.795288 (PMC8843038; doi:10.3389/fpls.2021.795288)
Supplement: Supplementary file 1 [file Data_Sheet_1.pdf]

Sup. Mat. 1

List of sources used in this study containing information on seeds consumed by waterfowl species


1. Allouche, L. (1983). Alimentation comparée du Canard Chipeau Anas strepera et de la Foulque Macroule Fulica atra pendant leur hivernage en Camargue. *Rapport Diplôme d'Études Approfondies, Montpelier, France*.

2. Amat, J.A. & Soriguer, R.C. (1982). Datos sobre selección de hábitat y ecología alimentícia del Porrón pardo (*Aythya nyroca*). *Acta Vertebrata* 388-394.

3. Bengtson, S. A. (1971). *Food and feeding of diving ducks breeding at Lake Mývatn, Iceland*.

4. Birger, S. B. G. (1907). *Über endozoische Samenverbreitung durch Vögel*.

5. Brochet, A. L., Mouronval, J. B., Aubry, P., Gauthier-Clerc, M., Green, A. J., Fritz, H., & Guillemain, M. (2012). Diet and feeding habitats of Camargue dabbling ducks: what has changed since the 1960s?. *Waterbirds*, *35*(4), 555-576.

6. Brouwer, G. A., & Tinbergen, L. (2002). De verspreiding der Kleine Zwanen, Cygnus b. bewickii Yarr., in de Zuiderzee, veer en na de verzoeting.

7. Danell, K., & Sjöberg, K. (1980). *Foods of wigeon, teal, mallard and pintail during the summer in a northern Swedish lake*. Swedish Sportsmen's Association.

8. De Vries, V. (1939). Bijdrage tot de voedselbiologie van een viertal eendensoorten, naar aanleiding van materiaal, afkomstig van Vlieland en Terschelling. *Limosa*, *12*, 87-98.

9. Figuerola, J., Green, A. J., & Santamaria, L. (2002). Comparative dispersal effectiveness of wigeongrass seeds by waterfowl wintering in south-west Spain: quantitative and qualitative aspects. *Journal of Ecology*, *90*(6), 989-1001.

10. Figuerola, J., Green, A. J., & Santamaria, L. (2003). Passive internal transport of aquatic organisms by waterfowl in Doñana, south-west Spain. *Global Ecology and Biogeography*, *12*(5), 427-436.

11. Fuentes, C., Sánchez, M. I., Selva, N., & Green, A. J. (2004). The diet of the Marbled Teal Marmaronetta angustirostris in southern Alicante, eastern Spain. *Rev. Ecol. Terre Vie*, *59*, 475-490.

12. Goyon Demonteil, M. C. (2004). Examen du contenu stomacal des canards sauvages de la Dombes: conséquences pour la gestion floristique des étangs. *Ecole Nationale Vétérinaire. Université Claude Bernard, Lyon*.

13. Green, A. J., & Selva, N. (2000). The diet of post-breeding Marbled Teal Marmaronetta angustirostris and Mallard Anas platyrhynchos in the Göksu Delta, Turkey.

14. Green, A. J., & Sánchez, M. I. (2003). Spatial and temporal variation in the diet of Marbled Teal Marmaronetta angustirostris in the Western Mediterranean. *Bird Study*, *50*(2), 153-160.

15. Green, A. J., Lovas-Kiss, Á., Stroud, R. A., Tierney, N., & Fox, A. D. (2018). Plant dispersal by Canada geese in Arctic Greenland. *Polar Research*, *37*(1), 1508268.

16. Guillemain M., Fritz H., Guillon N. & Simon G. (2002) Ecomorphology and coexistence in dabbling ducks: the role of lamellar density and body length in winter. Oikos 98, 547-551.

17. Hattermann, D., Bernhardt-Römermann, M., Otte, A., & Eckstein, R. L. (2019). Geese are overlooked dispersal vectors for vascular plants in archipelago environments. *Journal of Vegetation Science*, *30*(3), 533-541.

18. Hesselman, H.L. (1897). Några iakttagelser öfver växternas spridning. *Bot. Notiser* **97**, 97–112.

19. Höhn, E. O. (1948). The food and feeding movements of London mallards. *The London Bird Rep*, *12*, 36-38.

20. Jepsen, P. U. (1976). *Feeding ecology of Goldeneye (Bucephala clangula) during the wing-feather moult in Denmark*. Game Biology Station.

21. Jerling, L., Löfgren, A., & Lannek, J. (2001). Växtlivet i Stockholms skargård: mönster i tid och rum. *Sven Bot Tidskr*, 212-226.

22. Karmiris, I. L. I. A. S., Kazantzidis, S. A. V. A. S., & Papachristou, T. G. (2010). Variation in diet composition of wintering waterfowl among Greek wetlands. *Avocetta*, *34*(1), 21-28.

23. Kiss, J. B., Rekasi, J., & Sterbetz, I. (1984). A duna-deltában (Románia) élö tökés (Anas Platyrhynchos) ès cigányrécék (Aythya nyroca) táplálékának vizsgálata. *Puszta*, *2*, 39-51.

24. Kleyheeg, E., Klaassen, M., & Soons, M. B. (2016). Seed dispersal potential by wild mallard duck as estimated from digestive tract analysis. Freshwater biology, 61(10), 1746-1758.

25. Lanchon-Aubrais, E. (1992). *Conséquences d'un apport artificiel de nourriture sur le comportement d'un canard de surface: le Canard colvert(Anas platyrhynchos, L.) et d'un canard plongeur: le Fuligule milouin(Aythya ferina, L.) au lac de Grand-Lieu* (Doctoral dissertation).

26. Llorente, G. A., Ruiz, X., & Serra-Cobo, J. (1987). Alimentación otoñal de la Cerceta Común (Anas crecca) en el Delta del Ebro.

27. Lovas-Kiss, Á., Vizi, B., Vincze, O., Molnár V, A., & Green, A. J. (2018). Endozoochory of aquatic ferns and angiosperms by mallards in Central Europe. *Journal of Ecology*, *106*(4), 1714-1723.

28. Molodovsky, A. O. (1971). Feeding of Anas crecca L. and A. querquedula L. of the Gorky reservoirs. *Biol Nauki*, *11*, 20-25.

29. Mouronval, J. B., Guillemain, M., Canny, A., & Poirier, F. (2013). Diet of non-breeding wildfowl Anatidae and Coot Fulica atra on the Perthois gravel pits,

northeast France. *Wildfowl*, *57*(57), 68-97. (This data can also be found in this article)

30. Nilsson, L. (1972). Local distribution, food choice and food consumption of diving ducks on a South Swedish lake. *Oikos*, 82-91.

31. Olney, P. J. S. (1963). The food and feeding habits of Tufted Duck Aythya fuligula. *Ibis*, *105*(1), 55-62.

32. Olney, P.J.S. (1963). The food and feeding habits of teal *Anas crecca crecca* L. *Proc. Zool. Soc. Lond.* **140**, 169–210.

33. Olney, P.J.S. (1965). The autumn and winter feeding biology of certain sympatric ducks. *Trans. Int. Union Game Biol.* **6**, 309–320.

34. Olney, P. J. S. (1965). The food and feeding habits of Shelduck Tadorna tadorna. *Ibis*, *107*(4), 527-532.

35. Olney, P. J. S. (1968). The food and feeding-habits of the Pochard, Aythya ferina. *Biological Conservation*, *1*(1), 71-76.

36. Olney, P. J. S., & Mills, D. H. (1963). The food and feeding habits of goldeneye Bucephala clangula in Great Britain. *Ibis*, *105*(3), 293-300.

37. Owen, M., & Cadbury, C. J. (1975). The ecology and mortality of swans at the Ouse Washes, England. *Wildfowl*, *26*(26), 31-42.

38. Pehrsson, O. (1976). Food and feeding grounds of the Goldeneye Bucephala clangula (L.) on the Swedish west coast. *Ornis Scandinavica*, 91-112.

39. Pirkola, M.K. (1966). On the feeding habits of the mallard (*A. pl*) as revealed by crop and gizzard samples. *Suom. Riista* **18**, 67–81.

40. Pirot, J.Y. (1981). Partage alimentaire et spatial des zones humides camarguaises par 5 espèces de canards en hivernage et en transit. PhD thesis, Université Pierre et Marie Curie at Paris.

41. PIROT, J. Y., & PONT, D. (1987). Le canard souchet (Anas clypeata L.) hivernant en Camargue: alimentation, comportement et dispersion nocturne.

42. Player, P. V. (1971). Food and feeding habits of the Common Eider at Seafield, Edinburgh, in winter. *Wildfowl*, *22*(22), 100-106.

43. Rodrigues, D., Figueiredo, M. & Fabiao, A. (2002). Mallard (*Anas platyrhynchos*) summer diet in central Portugal rice-fields. *Game Wildl. Sc.* **19**, 55–62.

44. Sanchez, M.I., Green, A.J. & Dolz J.C. (2000). The diets of the White-headed Duck *Oxyura leucocephala*, Ruddy Duck *O. jamaicensis* and their hybrids from Spain. *Bird Study* **47,** 275-284.

45. Spärck, R. (1947). Tio års viltbiologiska undersökningar I Danmark. *Svensk Jakt* **85**, 287–292.

46. Schricke, V. (1983). Distribution spatio-temporelle des populations d'anatidés en transit et en hivernage en baie du Mont Saint-Michel, en relation avec les activités humaines. PhD thesis, University of Rennes I.

47. Srebrodol'skaya, N.I. & Pavluk, R.S. (1976). Nutrition of the Mallard *Anas platyrhynchos* in the western part of the Ukraine Polesye, USSR. *Vestn. Zool.* **2**, 78–80.

48. Staav, R. (1976). Gräsandens näringsval i en konstgjord sjö. *Zool. Rev.* **38**, 11–18.

49. Stempniewicz, L. E. C. H. (1995). Feeding ecology of the Long-tailed Duck Clangula hyemalis wintering in the Gulf of Gdansk(southern Baltic Sea). *Ornis Svecica*, *5*(3), 133-142.

50. Sterbertz, I. (1969). Investigations on wild-ducks in the inundation area of the River Tisza. *Aquila* **76–77**, 141–143.

51. STERBETZ, I. (1968). The comparative feed-examination of Gargeney and Teal (Anas querquedula L. and Anas crecca L.). *Acta Zoologica Academiae Scientiarium Hungaricae*, *55*, 1-4.

52. Sterbetz, I. (1979). A nagy lilik (Anser albifrons) a kis lilik (Anser erythropus) és a vetési lúd (Anser fabalis) táplálkozási viszonyai Magyarországon. *Aquila*, *85*, 93-106.

53. Sterbetz, I. (1990). Variations in the habitat of the Lesser White-fronted Goose (Anser erythropus L., 1758) in Hungary. *Aquila*, *96*, 11-18.

54. Sterbetz, I. (1991). Adatok a Magyarországon védett úszóréce (Anas sp.) fajok táplálkozásához. *Aquila*, *98*, 37-45.

55. Suarez, R.C. & Urios, V. (1999). La contaminación por saturnismo en las aves acuáticas del Parque Natural de El Hondo y su relación con los hábitos alimenticios. *Humedales Mediterráneos* **1**, 83–90.

56. Takács A; Molnár VA; Horváth O; Sramkó G; Popiela A; Mesterházy A; Lovas-Kiss Á; Green AJ; Löki V; Nagy T; Lukács BA (2017) The rare aquatic angiosperm Elatine gussonei (Elatinaceae) is more widely distributed than previously thought. - AQUATIC BOTANY 141, 47-50.

57. Tamisier, A. (1971). Régime alimentaire des sarcelles d'hiver Anas crecca L. en Camargue. *Alauda*, *39*, 261-311.

58. Tiussa, J. (1972). The autumn food of mallards, wigeon and teal during the hunting season. *Suom. Riista* **24**, 40–46.

59. Thomas, G.J. (1981). Field feeding by dabbling ducks around the Ouse Washes, England. *Wildfowl* **32**, 69–78.

60. Thomas, G.J. (1982). Autumn and winter feeding ecology of waterfowl at the Ouse Washes, England. *Zool. Soc. Lond.* **197**, 131–172.

61. Tóth, K., Bogyó, D., & Valkó, O. (2016). Endozoochorous seed dispersal potential of grey geese Anser spp. in Hortobágy National Park, Hungary. *Plant Ecology*, *217*(8), 1015-1024.

62. Viain, A., Corre, F., Delaporte, P., Joyeux, E., & Bocher, P. (2011). Numbers, diet and feeding methods of Common Shelduck Tadorna tadorna wintering in the estuarine bays of Aiguillon and Marennes-Oléron, western France. *Wildfowl*, *61*, 121-141.

63. Walmsley, J. G., & Moser, M. E. (1981). The winter food and feeding habits of Shelduck in the Camargue, France. *Wildfowl*, *32*(32), 99-106.
