# Supplementary material for: Functional Traits Drive Dispersal Interactions Between European Waterfowl and Seeds
Source: Front Plant Sci. 2022 Jan 31;12:795288. doi: 10.3389/fpls.2021.795288 (PMC8843038; doi:10.3389/fpls.2021.795288)
Supplement: Supplementary file 3 [file Data_Sheet_3.pdf]

Sup. Mat. 3

Trait values for the European Anatidae species for which diet studies containing identified seeds were found.

Species	Bill: Total Culmen	Bill: tip to Nares	Bill: Width	Bill: Depth	Tarsus Length	Mass	Feeding group
Anas acuta	43.175	31.925	13.375	14.85	35.025	944.62	Omnivore dabbling ducks
Spatula clypeata	63.35	47.9	18.175	18.375	31.4	612.56	Omnivore dabbling ducks
Anas crecca	35.075	29.1625	11.8625	11.45	25.9125	341.89	Omnivore dabbling ducks
Mareca penelope	37.35	26.475	15.325	12.425	33.875	770.03	Omnivore dabbling ducks
Anas platyrhynchos	56.175	40.975	20.75	17.825	39.65	843.42	Omnivore dabbling ducks
Spatula querquedula	37.15	29.975	14.2	12.75	24.575	325.6	Omnivore dabbling ducks
Mareca strepera	45.425	34.725	16.625	13.1	36.325	915.58	Omnivore dabbling ducks
Anser albifrons	46.575	27.65	18.175	17.225	64.2	2506.39	Terrestrial plant eaters
Anser anser	58.125	31.75	22.225	20.65	74.925	3302.41	Terrestrial plant eaters
Anser brachyrhynchus	46.875	26.2	18.525	14.975	73.15	2642.04	Terrestrial plant eaters
Anser erythropus	37.05	21.575	15.5	13.45	61.55	1755.5	Terrestrial plant eaters
Anser fabalis	73	40.2875	22.25	23.3875	77.9125	2754.73	Terrestrial plant eaters
Aythya ferina	47.1	30.5	17.6	13.55	31.575	822.99	Omnivore diving ducks
Aythya fuligula	39.6	27.65	19.25	13.225	28.475	701.17	Omnivore diving ducks
Aythya marila	44.1	29.675	23	14.175	32.275	1005.37	Omnivore diving ducks
Aythya nyroca	38.95	27.5	18.975	12.825	26.975	574	Omnivore diving ducks
Branta canadensis	50	28.2	19.9	15.3	73.3	2811.7	Terrestrial plant eaters
Branta leucopsis	39	20.3	16.1	11.7	64.3	1684	Terrestrial plant eaters
Bucephala clangula	34.325	17.725	15.8	11.1	34.9	918.56	Omnivore diving ducks
Bucephala islandica	34.65	18.25	16.65	12.75	37.875	921.21	Omnivore diving ducks
Clangula hyemalis	33.7	19.175	16.075	11.425	30.85	871	Marine invertebrate eating diving ducks
Cygnus columbianus	100.575	41.125	30.4	20.35	93.8	6377.96	Aquatic plant eaters
Cygnus cygnus	102.4	45	29.7	21.7	102	9350	Aquatic plant eaters
Cygnus olor	97.675	56.7	31.425	26.65	93	10682.04	Aquatic plant eaters
Marmaronetta angustirostris	43.95	36	14.925	13.175	31.85	477	Omnivore dabbling ducks
Melanitta nigra	45.925	24.6	21.4625	12.34	38.1875	1049.98	Marine invertebrate eating diving ducks
Mergus serrator	62.575	42.75	8.2	10.675	47.05	1015.17	Fish eating diving duck
Netta rufina	47.975	33.4	21.425	15.525	36	1118	Omnivore diving ducks
Oxyura leucocephala	45.76	28.5	22.98	14.1	36.08	687	Omnivore diving ducks
Somateria mollissima	51.125	27.975	19.225	16.575	44.4	2060.93	Marine invertebrate eating diving ducks
Tadorna tadorna	51.55	30.975	17.975	14.5	47.95	1146.83	Omnivore dabbling ducks
