# Supplementary material for: Functional Traits Drive Dispersal Interactions Between European Waterfowl and Seeds
Source: Front Plant Sci. 2022 Jan 31;12:795288. doi: 10.3389/fpls.2021.795288 (PMC8843038; doi:10.3389/fpls.2021.795288)
Supplement: Supplementary file 5 [file Data_Sheet_5.pdf]

Sup. Mat. 5.

Details on the RLQ and fourth-corner protocol.

The RLQ multivariate analysis technique requires three tables: i) the ***L*** table, which describes the occurrence of *p* species (in columns, i.e. our seed species) at *n* sampling sites (in rows, i.e. our waterfowl species), ii) the R table, which includes *m* environmental variables (in columns, i.e. our waterfowl traits) for *n* sampling sites (in rows, i.e. our waterfowl species), and iii) the Q table, which details *s* functional traits (in columns, i.e. our plant traits) for *p* species (in rows, i.e. our plant species).

Firstly, dimensionality was reduced in each of the tables separately with different ordination methods, allowing better capture of the variability of each dataset. We used the *dudi.hillsmith* function to ordinate the R and Q tables, which contained a mix of quantitative and categorical variables, with Hill Smith Principal Component Analyses (PCA; Hill & Smith 1976). For the presence-absence L table, we used the function *dudi.coa* to calculate a correspondence analysis. We then performed the RLQ ordination with the function *rlq*, selecting the axes that maximized the covariance between the linear combinations of the traits in the R and Q tables. The covariance between tables R and Q was higher for the first axis (99.04%) than the second axis (34.91%), but correlations for both axes were quite low (axis 1 = 0.31, axis 2 = 0.22). The RLQ accounted for 81% of the variability explained by the first two axes of the R table, and 84% of the variability explained by the first two axes of the Q table (see Table below).

We tested the overall significance of the RLQ analysis using the permutation function *randtest*. Furthermore, fourth-corner tests were applied to test the significance of the waterfowl trait x plant trait relationships in two ways. First, a fourth-corner test was applied directly to the raw R, L and Q tables with the function *fourthcorner*, to test for the significance of direct trait-trait relationships. Next, a fourth-corner test was applied to the correlations found between species traits and the first two RLQ axes, using the function *fourthcorner.rlq*. We used the permutation model 6 as an argument in all functions.

Total Inertia: 1.131				
Eigenvalues:				
Ax1	Ax2	Ax3	Ax4	Ax5
0.91	0.12	0.05	0.03	0.01
Projected inertia (%):				
Ax1	Ax2	Ax3	Ax4	Ax5
80.76	10.93	4.03	2.64	0.92
Cumulative projected inertia (%):				
Ax1	Ax1:2	Ax1:3	Ax1:4	Ax1:5
80.77	91.70	95.74	98.38	99.30
Eigenvalues decomposition:				

	eig	covar	sdR	sdQ	corr
1	0.91	0.95	1.96	1.61	0.30
2	0.12	0.35	1.22	1.29	0.22
Inertia & coinertia R (R):					
	inertia	max	ratio		
1	3.85	5.02	0.77		
12	5.35	6.74	0.79		
Inertia & coinertia Q (Q):					
	inertia	max	ratio		
1	2.61	2.93	0.89		
12	4.28	5.01	0.85		
Correlation L (L):					
	corr	max	ratio		
1	0.30	0.81	0.37		
2	0.22	0.78	0.28		

Table. Summary of the RLQ analysis.

References

Hill, M.O., and Smith, A.J.E. (1976). Principal component analysis of taxonomic data with multi-state discrete characters. Taxon 25, 249–255.
