# Supplementary material for: Functional Traits Drive Dispersal Interactions Between European Waterfowl and Seeds
Source: Front Plant Sci. 2022 Jan 31;12:795288. doi: 10.3389/fpls.2021.795288 (PMC8843038; doi:10.3389/fpls.2021.795288)
Supplement: Supplementary file 6 [file Data_Sheet_6.pdf]

Sup. Mat. 6.

Correlations between species traits and the first two axes of the RLQ analysis.

	Axis 1	Axis 2
Bill length from culmen	-0.802	-0.35
Bill length from nares	-0.383	0.186
Bill width	-0.769	-0.553
Bill depth	**-0.888**	-0.291
Tarsus length	**-0.957**	-0.545
Mass	-0.868	-0.601
Aquatic plant eaters	-3.184	-3.037
Fish eating diving duck	0.002	-2.816
Marine invertebrate eating diving ducks	0.198	-3.51
Omnivore dabbling ducks	0.307	**0.596**
Omnivore diving ducks	**0.981**	**-1.756**
Terrestrial plant eaters	**-1.752**	-0.907

Table 1. Correlations between each waterfowl trait and the first two axes of the RLQ analysis. Correlations considered as significant by the fourth-corner test are shown in bold.

	Axis 1	Axis 2
EIV for nutrients (N)	0.346	0.348
EIV for soil moisture (F)	**0.874**	-0.316
EIV for salinity (S)	0.217	0.606
EIV for temperature (T)	0.263	**0.624**
EIV for light exposure (L)	-0.089	**0.609**
Roundness	0.014	-0.006
Seed size (mm^3)	0.079	-0.124
Seed mass (mg)	0.072	-0.078
Seed density	0.023	0.200
Helophytes	0.808	1.273
Hydato-helophytes	0.719	0.458

Hydrophytes	**1.225**	-0.648
Hygrophytes	-0.038	0.131
Terrestrial	**-0.833**	0.296
Submerged	**0.607**	-0.339
Floating leaved	**0.526**	-0.3
Emerged	**0.585**	-0.137
Wet soils	0.08	-0.147

Table 2. Correlations between each plant/seed trait and the first two axes of the RLQ analysis. Correlations considered as significant by the fourth-corner test are shown in bold.
