# Supplementary material for: Functional Traits Drive Dispersal Interactions Between European Waterfowl and Seeds
Source: Front Plant Sci. 2022 Jan 31;12:795288. doi: 10.3389/fpls.2021.795288 (PMC8843038; doi:10.3389/fpls.2021.795288)
Supplement: Supplementary file 7 [file Data_Sheet_7.pdf]

Sup. Mat. 7

Results of the model selection procedure. Zero-truncated GAMs were fitted on the number of waterfowl species that consumed each seed species (hereafter number of vector species) against the predictor variables of soil moisture, nutrient, salinity, temperature and light exposure EIVs, and seed roundness, mass and density.

Model	ΔAIC
EIV T + EIV F + s(EIV N) + s(EIV S) + s(Seed mass) + s(Seed density) + s(Roundness)	0
EIV T + EIV F + s(EIV N) + s(EIV S) + s(Seed mass) + s(Seed density)	0.22
EIV T + EIV L + EIV F + s(EIV N) + s(EIV S) + s(Seed mass) + s(Seed density) + s(Roundness)	1.52
EIV T + EIV L + EIV F + s(EIV N) + s(EIV S) + s(Seed mass) + s(Seed density)	1.74
EIV F + s(EIV N) + s(EIV S) + s(Seed mass) + s(Seed density) + s(Roundness)	2.05
EIV T + EIV F + s(EIV N) + s(EIV S) + s(Seed mass) + s(Seed density)	2.47
EIV L + EIV F + s(EIV N) + s(EIV S) + s(Seed mass) + s(Seed density) + s(Roundness)	3.08
EIV T + EIV F + s(EIV N) + s(EIV S) + s(Seed mass) + s(Seed density)	3.43
EIV F + s(EIV S) + s(Seed mass) + s(Seed density) + s(Roundness)	4.48
EIV T + EIV F + s(EIV S) + s(Seed mass) + s(Seed density) + s(Roundness)	4.62
EIV T + EIV F + s(EIV S) + s(Seed mass) + s(Seed density)	4.83
**EIV F + s(EIV S) + s(Seed mass) + s(Seed density)**	**4.92**
EIV L + EIV F + s(EIV S) + s(Seed mass) + s(Seed density) + s(Roundness)	5.22
EIV L + EIV F + s(EIV N) + s(EIV S) + s(Seed mass) + s(Seed density)	5.48
EIV T + EIV L + EIV F + s(EIV S) + s(Seed mass) + s(Seed density) + s(Roundness)	5.62
EIV T + EIV L + EIV F + s(EIV S) + s(Seed mass) + s(Seed density)	5.7
Null model	34.6

Table 1. Fitted models with ΔAIC < 6 and null model ΔAIC. The selected final model is highlighted in bold.

	Coefficient	Standard error	Chi squared	P (Chi)
Intercept 1	-0.9568	0.2694		
Intercept 2	-0.0125	0.2881		
EIV F	0.1666	0.0273		
s(EIV S)	0.1047	0.0317	23.4	<0.0001
s(Seed mass)	0.1045	0.0471	13.58	0.003
s(Seed density)	0.0607	0.0884	22.11	<0.0001

Table 2. Coefficients and standard errors of the seed/plant traits used as predictors of the number of waterfowl species consuming each seed in the final model and results of the chi-squared tests for linearity of each predictor.
