# Supplementary material for: Functional Traits Drive Dispersal Interactions Between European Waterfowl and Seeds
Source: Front Plant Sci. 2022 Jan 31;12:795288. doi: 10.3389/fpls.2021.795288 (PMC8843038; doi:10.3389/fpls.2021.795288)
Supplement: Supplementary file 8 [file Data_Sheet_8.pdf]

Sup. Mat. 8

Sources for the Photos

No changes were made to any of the photos.
Photos of *Ceratophyllum demersum*, *Hippuris vulgaris* and *Najas marina* were taken by Lukács Balázs András.
Photos of other species were sourced from:

Andreas Rockstein. “Schoenoplectur lacustris” 25 Jul 2018. Online Image. Flickr. 12 Oct 2021. <https://www.flickr.com/photos/74738817@N07/42789104715/in/photolist-KTc8XD-28c8iMZ-29uLZWQ-28c8m5K-9k2nQY-hazXwg-2jsizHg-2jsmm75-2cPPUA-cXgwJW-2jsmmp4-2jsmmiC-2jsnEjm-WwHDEE-wFXSux-w2rdi5-CJzkzy>

Forest and Kim Starr. “starr-030612-0131-Bolboschoenus_maritimus-habit-Kanaha_Pond-Maui” 12 Jun 2003. Online Image. Flickr. 12 Oct 2021. <https://www.flickr.com/people/starr-environmental/https://www.flickr.com/photos/starr-environmental/24635396795/in/photolist-DwWRbB-CVQvTW-6fpYxv-DGpsBN-L5pqV4-2bPbB8y-DAjP94-xRpEzX-2jFR6by-2bPbBwj-EoaEUq-2cQMyvo-EnBmJw-EheUyx-EcFcuY-FWBQNr-FUiFdU-F8dn2z-UPXpMp-DimcZT-2j9f7NZ-UPXoaX-2hfLE82-JKnguM-LTJUe7-FCmTD3-MhoEHs-Dxioqv-6fpYxH-D7ctrt-DVo9pj-2hfLhNJ-E2J967-DFMn8g-2hfN7zb-5SsW6U-2hfNXrW-CV6QWK-2hfPbxk-2hfLo29-5SoB4r-DqcAik-2hfNaic-2hfLwi4-2iQ2k1d-5SsVZA-EetYXy-RxufmD-5UMnfN-UyEQQm]>

Forest and Kim Starr. “starr-120719-9483-Ruppia_maritima-habit_in_ponds-Hilo_Bay_Liliuokalani_Park-Hawaii” 19 Jul 2012. Online Image. Flickr. 12 Oct 2021. <https://www.flickr.com/photos/starr-environmental/24822070519/in/photolist-CpKxFr-EkBbCb-CNDd1e-CFgcfN-DPrAM6-CFnJ3M-CFnHBg-DbDYA5-CFgdy9-bWapRh-DsmSSS-D5higk-DCLrex-Duvrma-KiTSKn-cXg4bw-ak9EZp-yMF4u5-6cCaNj-itmLw1-5p9XRC-Jrtu2e-M92LKX-N4wuZx-LYcUXW>

FortBienVert. “Salicorne [Salicornia europaea]” 26 Jun 2006. Online Image. Flickr. 12 Oct 2021. <https://www.flickr.com/photos/47689195@N00/293924476/in/photolist-rYrvo-MVFing-xgShpM-8hEGMv-2bBm993-pbMgEi-2jyoger-epGjuq-2e4XxtY-MH1tLM-56zGNN-28RGhpw-2jyognT-wE2X5-oPMfZ1-RBqG6E-kfFvou-6nTrbF-NPUBUH-2jyszAs-2jyszyP-Nmu2Jm-M6EhFo-N4mHtF-oe385D-M6EhJ9-2jKLJem-U3h4iK-N4mHkK-z1Ci1j-sBs3Q5-p7f18E-NAn1pM-28m7fNh-ptQUAp-8XZNyD-jjp6L-cGdfDs-x1qGcM-dpyEVA-9Z8k8B-a7quih-2jKLJad-4yBVx8-sn9PF9-UC3bCS-dfJcs8-peVod-23uWBym-kK4uVV>

Gertjan van Noord. “Muizenstaart - Myosurus minimus” 15 Apr 2021. Online Image. Flickr. 12 Oct 2021. <https://www.flickr.com/photos/gertjanvannoord/51118905666/in/photolist-SyUU3b-2kTcKGw-2kRSWoK-2iXzZew-2ky5mcK-KgQHUf-abN9p3-pAtWre-2kS6nzT-2kRSWEr-2iXAhvR-2iPbUrT-2iUCZFv-2j96HwT-2j96HnK-2j27zeS-2iUFJan-2iUCZpy-2iUFHPn-2iNUk4s-2iNRCKs-2iVrYNP-pTDYg4>

Joan Simon. “Setaria viridis” 28 Sep 2014. Online Image. Flickr. 12 Oct 2021. <https://www.flickr.com/photos/simonjoan/15447781336/in/photolist-px4Vcw-px4TdS-6P32ib-6NXSaX-7bnzHK-6NXRHc-6NXStF-6P31Q9-6P321Y-2m8PPAD-8zZzHU-A4SiHw-8CScHd-8zYb11-8AxCkD-2jr6Gy9-2jr6Gub-2jr6Gwv-Y1TAhk-Y1TAje-7brnYW-Y1TAo2-zgK5sx-ae6TUT-m83N77-2dispJS-VcCFyu-vTHfuX-QMdi46-9UsiCt-Aax7hP-oowmUQ-YDnRsW-5kGGF9-apGbAN-

5kCqVX-5kCrmg-242zphJ-2g7GDHs-v3Rzdc-v3zavD-PBNGy4-v35tYW-v3z8Qp-v3zguT-v1fG67-uL82XK-7gnuie-u6ysWG-uL7Vb8>

Matt Lavin. “Eleocharis palustris” 25 Jun 2016. Online Image. Flickr. 12 Oct 2021. <https://www.flickr.com/photos/plant_diversity/28639516472/in/photolist-KCM1sL-2kVtPmF-wThHpk-cWr2fh-2gxgDkH-2gxgDt3-cWr3eQ-dfD2yE-dfCXXr-dfCYFH-2gxgDfc-dfCYMP-cWr3cS-cWr37s-2gxh3v1-cWr1pQ-2hNFBJW-2jtpja3-pAu9LK-pjgjEy-LaAVYc-L69Jey-pRJoYE-pRJoPb-UPSUjr-bAnNjH-ae9G9Y-5Ee9SD-g3Vdie-ZKPybS-UzzppY-cWBpLf-eV6iav-nK2iJk-eV6i2p-eVhFxY-KuCswC-eVhErW-f1mWvu-2hxvWie-2hxvWjG-2hxvWkJ-2jtpjhh-2jtpjcN-2jtnZRF-2hxwW6z-29YkaAq-2mgwEHC-2jtnZMh-26G2GC7>

SimonLH64. “Greater Plantain” 20 May 2016. Online Image. Flickr. 12 Oct 2021. <https://www.flickr.com/photos/137246163@N04/26531795083/in/photolist-GqwojP-2kePdog-2m5p4jx-2m5q5tr-2m5knBU-2m5q3rR-2m5usi7-v9WFbf-2iCM6sP-CpXYxf-JD19tY-8uughQ-cR6it7-8b3mEH-gEL7XV-bzjpqC-2iCNvZi-8b3peH-CpY58y-bNe42k-nHmK5r-C22yWp-2jzQBtM-TAkPYP-2iCNw4r-8b6E5u-29fLPep-MBrbw6-HhXqLf-8b3orz-fEaRJs-2iCNvXE-TxqzN3-fPxj44-C8oTm3-2fSzmzN-2iCJqhk-TAkPDv-2fSzmAQ-Cse8NM-CpXBNu-CseqjH-cR6hyY-2eKqnim-2hY4mZu-2hY7Wmn-yJm9Xf-2hY7Wjy-2hEX8As-JZDD5a>
